# Bridging Ayurveda with evidence-based scientific approaches in medicine

**DOI:** 10.1186/1878-5085-5-19

**Published:** 2014-11-01

**Authors:** Bhushan Patwardhan

**Affiliations:** Interdisciplinary School of Health Sciences, Savitribai Phule Pune University, Ganeshkhind, Pune, Maharashtra 411007 India

**Keywords:** Ayurveda, Traditional, complementary and integrative medicine, Epistemology, Evidence-based medicine, predictive, preventive and personalized medicine, PPPM

## Abstract

This article reviews contemporary approaches for bridging Ayurveda with evidence-based medicine. In doing so, the author presents a pragmatic assessment of quality, methodology and extent of scientific research in Ayurvedic medicine. The article discusses the meaning of evidence and indicates the need to adopt epistemologically sensitive methods and rigorous experimentation using modern science. The author critically analyzes the status of Ayurvedic medicine based on personal observations, peer interactions and published research. This review article concludes that traditional knowledge systems like Ayurveda and modern scientific evidence-based medicine should be integrated. The author advocates that Ayurvedic researchers should develop strategic collaborations with innovative initiatives like ‘Horizon 2020’ involving predictive, preventive and personalized medicine (PPPM).

## Review

Ayurveda is one of the traditional systems of medicine that practices holistic principles primarily focused on personalized health. Originated in India, Ayurveda is one of the ancient yet living health traditions. Ayurveda is commonly referred as ‘science of life’ because the Sanskrit meaning of *Ayu* is life and *Veda* is science or knowledge. *Charaka Samhita*, *Sushruta Samhita* (~400 BC–200 AD) *and Ashtanga Hridaya of Vagbhata* are main classics, which give detailed descriptions of over 700 herbs and 6,000 formulations. *Madhav Nidan* (~800 AD), a diagnostic classic, provides over 5,000 signs and symptoms. Life in Ayurveda is conceived as the union of the body, senses, mind and spirit. The concept of *Prakriti* or individual nature has a central role in Ayurveda therapeutics. With over 400,000 registered Ayurveda practitioners, the government of India has a formal structure to regulate its quality, education and practice.

Prolonged use of Ayurveda by people has also led to several home remedies for common ailments. Ayurvedic medicines contain sophisticated therapeutic formulations. Ayurveda is also a person-centered medicine (PCM), which deals with healthy lifestyle, health promotion and sustenance, disease prevention, diagnosis and treatment [1]. The holistic concepts of Ayurveda give emphasis to health promotion, disease prevention, early diagnosis and personalized treatment. There seem to be substantial similarities between the traditional systems like Ayurveda and the innovative approach of predictive, preventive and personalized medicine (PPPM) [2]. The Horizon 2020 initiative of the European Union rightly considers PPPM as the hardcore of its strategy [3].

The need for scientific evaluation of Ayurveda has been recognized for a long time [4]. Ayurveda has personalized approach involving constitutional assessment, which can guide primary prevention, diagnosis and therapeutics. Ayurveda also offers detailed guidance about food, nutrition and diet as per the individual constitution or *Prakriti* as well as seasons [5]. The scientific value of basic principles of Ayurveda like *Prakriti* is being studied in context to biology and genomics [6].

Ayurveda as an ancient science of life has a long history, and its basic principles may be valid even today. However, essence of any science is a continuous quest for new knowledge through research, development and newer applications. The mode of manifestation of disease has changed. The geo-climatic environment, plants, animals and microbes have changed. Human behavior, lifestyle and genetics have changed. Clearly, classical Ayurveda of yesteryears cannot be blindly practiced without contemporary modifications. Continuous research on safety, quality and efficacy of Ayurvedic drugs and procedures is needed. Systematic documentation and critical analysis of clinical practice are necessary. Sanskrit savvy scholars from India should not be considered as the only custodians of knowledge and practice of Ayurveda. Several Western scholars like Meulenbeld have contributed to emergence of the new Ayurveda [7]. Many countries in the world especially Germany, Italy, Hungary, Switzerland, United States have institutions where Ayurveda is correctly practiced with respect to traditions and high professional competence [8, 9].

Ayurveda was meant to be open for new ideas, principles and knowledge for continuous and systematic progress. However, its progression seems to be stalled during the last several centuries resulting in chronic stagnancy of today. Heritage pride and past glory-based emotional attitudes seem to be predominant among practitioners as against evidence-based quest of scientific research. There seem to be an evident complacency, defensive and dogmatic attitude and often pure sentimentalism rather than a pragmatic scientific outlook. We need to recognize that emotions, experience and evidence are not mutually exclusive. Becoming modern is not a crime; it does not prevent anyone from maintaining cultural identity. No tradition is a static entity; modernity results from evolving traditions. For instance, Charaka would not have ignored technologies like electron microscope if they had been available during his time [10]. While accepting modern tools and technologies, it is equally important to respect epistemological value of knowledge system like Ayurveda. Embracing modernity by Ayurvedic community does not mean blind acceptance of Western logic and reductive methodologies. In fact, increased recognition to disciplines like systems biology is indicative of modern science moving towards holistic concepts. Therefore, this may be the opportune time to facilitate integration of Ayurveda, Western biomedicine and modern science.

Recently, many experts and critiques have raised concerns that while the popularity of traditional and complementary medicine (T&CM) is growing, this sector is still grappling to discover appropriate models and demonstrate sufficient scientific evidence [11]. Ayurvedic medicine is no exception to these concerns. However, for understanding Ayurveda from modern terms, one also needs to understand its epistemology.

The objective of any medical research should be to assess health effects, minimize bias, chance effects and confounders. A well-designed rigorous scientific research on medicines and therapeutic practices of Ayurveda is necessary. The Ayurveda sector has to take cognizance of important initiatives like standards for reporting observational epidemiology (STROBE) and consolidated standards on reporting trial (CONSORT) in the methodological domain to develop epistemologically sensitive appropriate methods. Evidence-based Ayurveda needs appropriate blends of modern science, rigorous trial methods and observational studies. Arguably, the nature of evidence in case of Ayurveda may be different from that of Western biomedicine. The status of Ayurveda as an evidence-based medicine is also reviewed here.

### The evidence in right perspective

In philosophy, evidence is closely tied to epistemology, which considers the nature of knowledge and how it is acquired. Many proponents of T&CM sector argue that inability to measure something using present scientific methods is not a proof of its nonexistence. However, inability of measuring something is certainly not a proof of its existence.

It is also argued that future studies involving comparisons of T&CM systems with modern medicine need to be on the leveled playing field for evaluating outcomes from both an allopathic and a whole-system points of view [12]. Instead of any hierarchy of evidence, a circular model has been proposed to arrive at pragmatic but rigorous evidence which would provide significant assistance in clinical research [13]. Appreciably, over a period of time, traditional Chinese medicine (TCM) is starting to create large body of scientific evidence to support safety, pharmacology and clinical efficacy [14]. Ayurvedic medicine also needs to first discover epistemologically sensitive methods and then build objective scientific evidence with reasonable consistency to justify clinical decision making and therapeutics.

### Ayurveda epistemology

The epistemology of Ayurveda is based on the relation between microcosm and macrocosm involving five basic elements (*mahabhoota*), three dynamic principles similar to humors (*dosha*), seven types of tissues (*dhatus*) and many other unique concepts. An introduction to basic concepts may be useful for readers who are not familiar with epistemology of Ayurveda [15]. In general, Ayurveda is experiential, intuitive and holistic, whereas that of the modern medicine is based more on experimental, analytical and reductive reasoning. The relationship between Ayurveda and modern science is similar to the relationship between the ‘whole’ and the ‘parts’, where the sum of the parts need not be equal to the whole [16]. Modern medicine is based more on rationalism, reductionism with deeper understanding of molecules, cells, organs or diseases as parts. In the process, however, the sight of the whole person seems to have been somewhat neglected. Integrative, whole system approaches like PPPM and PCM as well as traditional and holistic systems like Ayurveda may need epistemologically sensitive research methodology.

Ayurveda is uniquely patient-oriented where the Ayurvedic physician diagnoses, treats and dispenses medicine to every individual patient. This important principle can form the basis for a form of personalized medicine which will give maximum therapeutic efficacy and high safety to a particular person with a particular disorder, under specified conditions depending on individual constitution, and properties of materials. *Prakriti* specific prescription may also include supportive therapies, diet and life-style advice so as to regain physiological balance, finally resulting in the removal of the disorder. A decision-support system known as AyuSoft (developed by Center for Development of Advance Computing and University of Pune, Ganeshkhind, Pune India) based on Ayurveda knowledge has been shown to be useful in determination of individual *Prakriti* and personalized treatments [17].

The conventional, experimental and diagnostic methods based on pathophysiology mostly rely on limited markers as evidence of health [18]. Applicability of such restrictive approaches to understand complex systems like Ayurveda has been questioned. Person-centered integrative medicine, which considers the whole person, needs new sets of experimental methodology. Holistic complex systems like Ayurveda may need approaches like the Bayesian theory rather than a classical statistical frequentist approach [19]; however, no serious experimental efforts have been made to test this hypothesis [20].

### Evidence-based medicine

Works of famous scientist Archie Cochrane on efficacy and effectiveness [21] and meta-analysis as a method of summarizing the results of randomized trials [22] have led to a powerful research and analysis tool in the form of ‘systematic reviews’, which empowered clinicians and researchers decision making. These efforts finally led to the evolution of evidence-based medicine (EBM) as a new approach to bring more rational and analytical evidence for research-backed practice of medicine [23]. The principles of EBM consider consistency of clinical practice quality and quality of scientific evidence to develop evidence-based practice.

In the following section, we have critically analyzed present situation regarding these two important aspects and have reviewed the status of Ayurveda as an evidence-based medicine.

### Evidence base for Ayurvedic medicine

It is very important to review available evidence in the right perspective. In case of Ayurveda, the evidence can be drawn from two main sources. First, source of evidence may be based on historical, classical and present nature of clinical practice. Here, the documentation of practice to support various claims is very crucial. Mere reference to classical texts is not sufficient as evidence for practice. The second source of evidence may be based on scientific research to support various theories, medicines and procedures used in Ayurvedic medicine. A critical situation analysis of present status of clinical practice and scientific research on Ayurvedic medicine may be necessary at this stage.

#### Clinical practice

Arguably, the clinical practice of classical Ayurveda is rare. Ayurvedic practitioners are reported to adopt allopathic practices for better acceptance in urban settings [24]. Although, huge knowledge resource and wisdom is available from many Ayurveda classic books, systematic data on actual use and evidence of reproducible outcomes is not available in public domain. Standard treatment protocols for practitioners are not available. Systematic documentation and reliable data on pharmacoepidemiology and pharmacovigilance for clinical practice, safety and adverse drug reactions are not available as open access, although a modest beginning has been made [25]. The status of professional [26] and continuing education [27] as well as attitudes of practitioners towards safety [28] are also worrying. As per present regulations in India, no scientific or clinical data is required for manufacture and sale of classical Ayurvedic medicines. Technically, sound pharmacopoeia, good manufacturing practices, quality control and pharmaceutical technologies for Ayurvedic medicine are still evolving [29, 30]. Issues related to appropriate research methodologies or treatment protocols for Ayurveda have also not been properly addressed. Many critiques are demanding better coordination between stakeholders, continuous dialogue with scientific community [31] and total overhaul of the curriculum and pedagogy along with the need for crosstalks between different streams [32]. Recent report on status of Indian medicine and folk healing indicates the need to strengthen research and use of Ayurveda, yoga, unani, siddha, homeopathy (AYUSH) systems in national health care [33]. The need for innovation is also urged by thought leaders in this sector [34]. In short, the evidence base to support good clinical practice, guidelines and documentation in Ayurvedic medicine remains scant and grossly inadequate.

#### Scientific evidence

Controlled clinical trials are taken as the highest level of evidence. Ayurveda lags far behind in scientific evidence in quantity and quality of randomized controlled clinical trials (RCTs) and systematic reviews. For instance, out of 7,864 systematic reviews in the Cochrane Library, Ayurveda has just one, while homeopathy and TCM have 5 and 14, respectively. Substantial grants have been allocated to ambitious national projects involving reputed laboratories. However, the design, methodology and quality of clinical trial on Ayurvedic medicines seem to lack the expected rigor [35]. Of course, this does not mean that the RCT model is suitable to clinical research in Ayurveda. RCTs have already been subjected to criticism [36]. Value of observational studies cannot be ignored. Certainly, there is a need to develop appropriate research methodology for complex whole system, whole-person-centered clinical trials as an alternative to RCTs. Already, scientists are advocating robust clinical study designs based on personalized approach and metabolomics with only one patient [37]. Thus, non-suitability of RCTs should not be used as an excuse for avoiding rigorous scientific research and clinical documentation.

Few noteworthy attempts related to research and practice include a national program on Ayurvedic biology [38, 39], Ayugenomics [40], whole systems clinical research [41–43], good clinical practices guidelines, digital helpline [44], decision support system AyuSoft, and systematic reporting standards on lines with CONSORT for Ayurveda [45, 46]. Recent efforts to develop robust clinical protocols for comparing effectiveness of complex Ayurvedic and conventional treatments are laudable [47]. Other notable efforts related to integrative therapy for leishmaniasis have been able to generate sufficient scientific evidence [48]. Agreeably, many of these efforts could not produce any remarkable products, processes or protocols, and desirable impact on a scientific community is yet to be seen. The need to enhance collaborative culture between Ayurvedic and modern scientific communities has been rightly stressed [49].

As a result, Ayurvedic medicine continues to remain subcritical in research publications, which is an important indicator of external evidence [50]. The present scientific evidence in support of Ayurvedic medicine remains extremely poor. The House of Lords and European Union have put several restrictions on Ayurvedic medicines [51]. Many articles lamenting poor quality of Ayurvedic medicines, presence of heavy metals and other safety compromising substances have been published [52, 53]. This situation may lead to further denigration, which can adversely impact the development of evidence base for Ayurveda.

#### Ayurvedic genomics and epigenomics

According to Ayurveda constructs, doshas are the dynamic principles, which govern a person’s physical, physiological and psychological functions including metabolism. Ayurveda describes three doshas namely vata, pitta, and kapha. The proportional domination of doshas in an individual is expressed as *Prakriti*, which broadly mean a body type or individual nature. An Ayurvedic physician determines the *Prakriti* of a patient so as to personalize treatment. The Ayurvedic description clearly suggests that the innate dispositions are represented by individual *Prakriti*, which represent phenotypes. Classifying humans based on phenotypes still remains a challenge to biomedical science. A number of research groups are now investigating the correlation between Ayurvedic phenotypes and individual human genotypes. A pioneering study showed significant correlation between HLA alleles and Ayurvedic *Prakriti* type [54]. Later, it was also hypothesized that different *Prakritis* may possess different drug metabolism rates associated with drug-metabolizing enzyme polymorphism. In another genotyping study, significant correlations between CYP2C19 genotypes and major classes of *Prakriti* types have been reported [55]. A project to study genomic variation analysis and gene expression profiling of human, *Prakriti* based on the principles of Ayurveda is underway. Now, it is hoped that going beyond genomics is necessary to understand how environment and behaviors can be responsible for inheritable changes when the genome remains unchanged. This science of epigenetics is seen as a future hope to get answers to many puzzles. It is felt that detailed understanding of Ayurvedic concepts like *Prakriti* may actually facilitate this process. However, no specific genotype has yet been specifically related to a *Prakriti* type.

#### Ayurvedic concept and predictive diagnosis

Modern biomedicine recognizes progressive nature of diseases like cancer and diabetes. It is known that slow yet progressive pathophysiological changes result in a transition from a healthy state to diseased state. Ayurvedic concept of *shatkriyakaal* elaborates a six-stage progressive transition from balanced to unbalanced stage leading to disease manifestation in a person. These six stages are unique and may help early recognition and early diagnosis much before onset of measurable clinical symptoms of diseases. It is possible to undertake a systematic cohort study by stratifying patients in the six categories. Each of the cohorts can be carefully followed up to study pathophysiological, genetic, and epigenetic and metabolomic differences. This may give leads towards the identification of new markers and early predictions, which can then be used for prevention and personalized treatments.

Thus, Ayurveda and PPPM concepts have many similarities, where both do not merely consider concept of disease in isolation but consider the diseased ‘person’. The need to define a common model of health and disease between the western and eastern knowledge systems has been pointed out earlier [56, 57]. Therefore, a collaborative project based on concepts of PPPM and Ayurveda may help to better understand disease progression and predictive diagnosis of diseases like cancer and diabetes. In this context, recent efforts to correlate traditional Ayurvedic and modern medical perspectives on cancer are very relevant. In a qualitative study, it was observed that Ayurvedic medicine offers a unique perspective on the biomedical diagnosis of cancer. Due emphasis on restoring wholeness, use of natural remedies focus on emotional health, and emphasis on prevention strategies were found to be unique features of Ayurvedic interventions [58].

### Moving towards evidence base

Several issues need to be addressed for Ayurveda to move towards acceptable evidence base. Concerns related to protocols, problems and potential of Ayurveda in context to evidence-based T&CM have been recently discussed [59]. Few critiques have opined that basic concepts of Ayurveda should not be distorted to suit convenience or availability of biomedical research models [60]. Arguably, prevailing pre-clinical methods and clinical models like RCTs may not be suitable to validate Ayurvedic medicine. However, the onus of developing suitable models to build necessary evidence must be voluntarily accepted by the Ayurveda sector. Some efforts in the direction to conduct the whole system clinical trials are already in progress [61].

A critical review and analysis indicate that the present Ayurvedic medicine is severely deficient in scientific evidence related to clinical practice and scientific research. Ayurveda sector needs to go beyond mere scholarly recitals, reviews and defensive interpretations, which are abundant in current literature. Ayurveda needs to be studied and experimented with help of new models based on modern science and biology. Ayurvedic medicine needs more rigorous scientific research for evaluating safety, quality and efficacy [62]. Many lessons learned in the past may guide our quest for evidence-based Ayurveda in the future [63]. It may be worthwhile to learn from collaborative research networks like IN-CAM from Canada [64] and CAMbrella from Europe [65]. Ayurveda sector needs to get connected with Indian and global scientific networks not as a bureaucratic process but for scientific and professional pursuits.

## Conclusions

This review and analysis is carried out with a caveat that the methods and evidence approach of biomedicine may not be directly applicable to Ayurveda. However, either Ayurveda has to discover its own methodology and approach for evidence or should face the critical analysis as per the conventional approach of EBM. Avoiding any critical appraisal under the pretext that it is a holistic system and that the present methods like randomized controlled trials are not applicable may not sustain for a long time.

**Figure 1:**
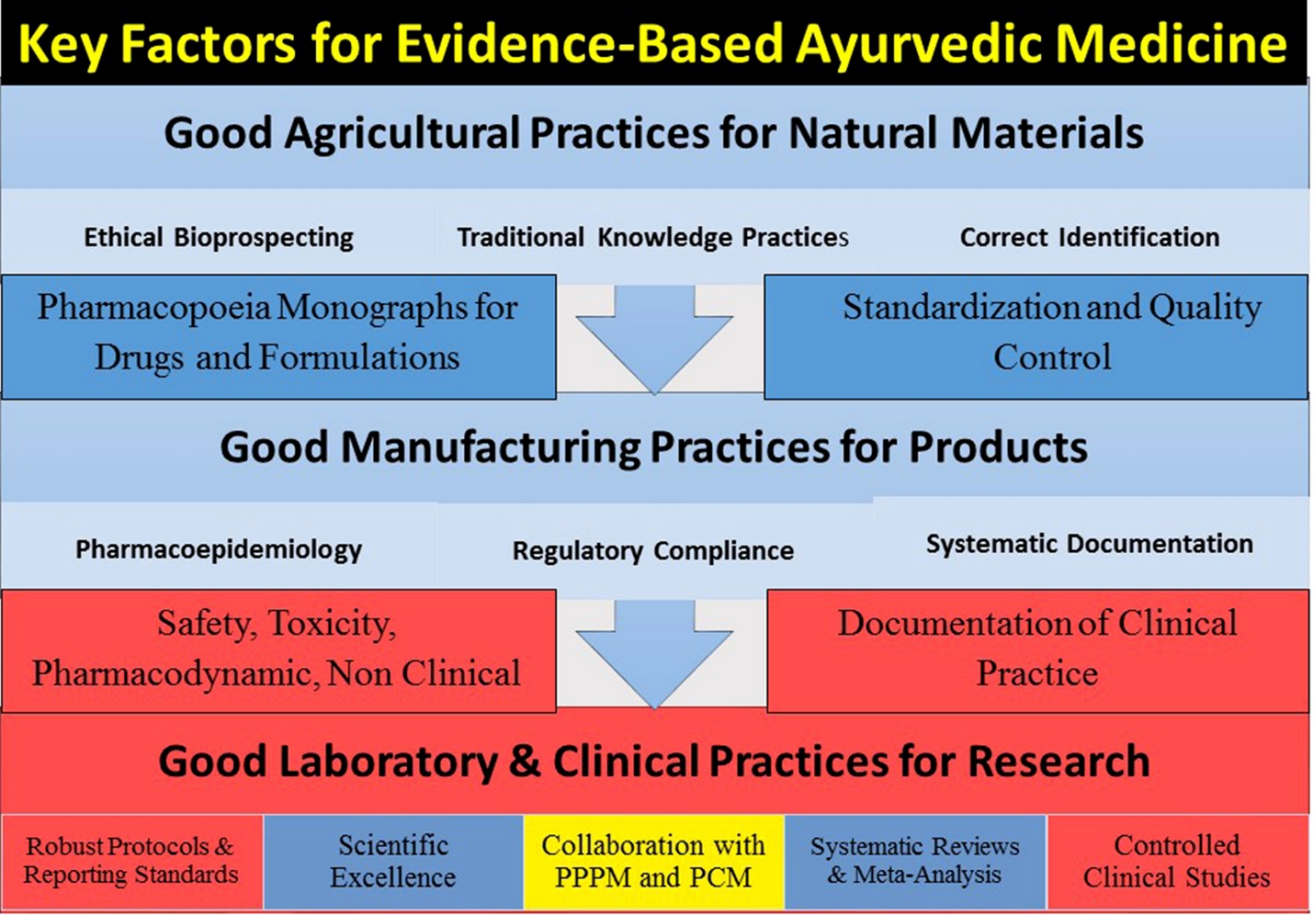
**Key factors for evidence-based Ayurvedic medicine.**

The Ayurvedic sector should urgently recognize and address the need for scientific evidence [66]. Systematic documentation, appropriate methodology and rigorous experimentation in accordance with good practices coupled with epistemologically sensitive approaches will remain crucial to move towards evidenced-based Ayurveda. Key factors crucial for Ayurveda to move towards evidence-based scientific approaches related to quality of drugs and practices are portrayed in Figure 1. The question of epistemologically sensitive methods is relevant only to biomedical laboratory and clinical research. The good agricultural practices for procurement of raw materials and good manufacturing practices for Ayurvedic drugs must be in accordance with the globally accepted norms.

### Integration of PPPM and Ayurveda

The new philosophy of health care is moving from illness to wellness, from treatment to prevention and early diagnostics and from generalized approach to personalized medicine. As discussed in this review, there are several similarities between the concepts of PPPM and Ayurveda. Search of novel models for integrative medicine indicates the need for collaborations between traditional systems like Ayurveda and contemporary western biomedicine [67]. Agreeably, many concepts from Ayurveda have not yet been validated with help of modern science. Therefore, it is felt that collaborative efforts between scientific researchers from Ayurveda and PPPM seem to be a mutually beneficial proposition. Such integration bringing the best of the western biomedicine and eastern traditional knowledge systems like Ayurveda may lead to high impact projects. Suitable industry collaborators can also be roped in from both respective regions. Thus, the three dimensional priority of ‘Horizon 2020’ can be effectively addressed through integration of PPPM and Ayurveda. Such integration certainly has contemporary significance and will help to address societal challenges presently faced in the global health care sector.
